# *Artemisia dracunculus* (Tarragon): A Review of Its Traditional Uses, Phytochemistry and Pharmacology

**DOI:** 10.3389/fphar.2021.653993

**Published:** 2021-04-13

**Authors:** Halina Ekiert, Joanna Świątkowska, Ewa Knut, Paweł Klin, Agnieszka Rzepiela, Michał Tomczyk, Agnieszka Szopa

**Affiliations:** ^1^ Chair and Department of Pharmaceutical Botany, Jagiellonian University, Medical College, Kraków, Poland; ^2^ Family Medicine Clinic, Medizinisches Versorgungszentrum (MVZ) Burgbernheim GmbH, Burgbernheim, Germany; ^3^ Museum of Pharmacy, Jagiellonian University, Medical College, Kraków, Poland; ^4^ Department of Pharmacognosy, Faculty of Pharmacy, Medical University of Białystok, Białystok, Poland

**Keywords:** tarragon, traditional medicine use, chemical composition, biological activity, potential medicinal value, position in cosmetology, biotechnological studies

## Abstract

*Artemisia dracunculus* L. (tarragon), Asteraceae, is a species that has long been used in traditional Asian medicine, mainly in Iran, Pakistan, Azerbaijan and India. It is known as a spice species in Asia, Europe and the Americas. The raw materials obtained from this species are herb and leaf. The presence of essential oil with a highly variable composition, as well as flavonoids, phenolic acids, coumarins and alkamides, determines the medicinal and/or spice properties of the plant. In traditional Asian medicine, this species is used, for example, in the treatment of digestive system diseases, as an analgesic, hypnotic, antiepileptic, anti-inflammatory and antipyretic agent, and as an effective remedy in the treatment of helminthiasis. Nowadays, *A. dracunculus* is the subject of professional phytochemical and pharmacological researches. Pharmacological studies have confirmed its anti-inflammatory and analgesic effects known from traditional uses; they have also proved very important new findings regarding its biological activity, such as antioxidant, immunomodulating and anti-tumour activities, as well as hepatoprotective and hypoglycaemic effects. *A. dracunculus* has long-held an established position in the food industry as a spice. And its use is growing in the cosmetics industry. Moreover, it is the subject of biotechnological research focused mainly on the development of micro-propagation protocols.

## Introduction

Over the last few years, there has been a noticeable increase in interest in phytochemical and pharmacological studies concerning various species of the genus *Artemisia* L (Asteraceae) (Tan et al., 1998; Willcox, 2009; Koul and Taak, 2017). This interest is undoubtedly due to the fact that the Nobel Prize in Physiology or Medicine 2015 was awarded for the discovery in *Artemisia annua* (annual mugwort) of artemisinin–a sesquiterpenoid lactone, and proving its effectiveness in the treatment of malaria (Długońska, 2015; Efferth et al., 2015). Among the subjects of research is *A. dracunculus*, a species native to Siberia and Mongolia. In Europe, this species is a popular spice plant; in Asian countries (Iran, Pakistan, Azerbaijan, India), this species has long been used in traditional medicine. It has been used both in the treatment of gastrointestinal diseases and as an anesthetic, hypnotic and anti-epileptic agent. It has been recommended as an effective treatment for inflammation, fever and helminthiasis (Aglarova et al., 2008; Bora and Sharma, 2011; Obolskiy et al., 2011).

Contemporary professional research has proven various important aspects of the biological activity of extracts from both the entire aerial part and/or leaves of this species, as well as from its essential oil. Its antibacterial, antifungal and antiprotozoal properties have been documented, together with its extremely valuable antioxidant, immunomodulatory and antineoplastic properties (Abtahi Froushani et al., 2016; Hassanzadeh et al., 2016; Bedini et al., 2017; Navarro-Salcedo et al., 2017; Mohammadi et al., 2020). These studies have also been proven to have hepatoprotective, hypoglycaemic and thyroid regulating effects (Méndez-Del Villar et al., 2016; Zarezade et al., 2018; Mohammadi et al., 2020). An antidepressant effect has also been documented (Wang et al., 2018). The anti-inflammatory and analgesic effects known from applications in traditional medicine have also been confirmed (Abtahi Froushani et al., 2016; Wang et al., 2018; Safari et al., 2019). Moreover, an examination of relevant professional research also shows that the position of *A. dracunculus* as a plant species with cosmetic properties is rising (Ribnicky et al., 2004; Yamada et al., 2011; Chaleshtori et al., 2013). According to modern research, tarragon appears not only to maintain its position as a valuable spice plant, but above all, as an important plant with potential medicinal and cosmetic properties.

The main goal of this review is to present the latest research on both the chemistry and new findings on the biological activity of *A. dracunculus*, proven by professional research. Earlier reviews by Aglarova et al. and Obolskiy et al. (Aglarova et al., 2008; Obolskiy et al., 2011) are quite generalized and don’t contain the latest, detailed information on this species, which is valuable in relation to pharmacology, cosmetology and food industries. In addition, the paper encompasses all previously known information concerning its biology and chemistry as well as the traditional medicine and culinary applications of the species under consideration.

## General Information on the Species

The name *Artemisia dracunculus* is derived from the Latin word “dracunculus” meaning “a small dragon”, and refers to the shape of the leaves, which resemble dragon tongues (Aglarova et al., 2008). *A. dracunculus* has numerous (about 50) Latin synonyms, including *Absinthium cernuum* Moench, *Achillea dracunculus* Hort., *Artemisia aromatica* A. Nelson, *A. cernua* Nutt., *A. crithmifolia* L., *A. dracunculiformis* Krasch., *A. dracunculina* S. Wats., *A. dracunculoides* Pursh (GBIF.org (2020); The Plant List, 2013; Catalogue of Life, 2020; Missouri Botanical Garden, 2020). The English names include tarragon, estragon, dragon sagewort, dragon wormwood, false tarragon, French tarragon, green sagewort, linear-leaved wormwood, Russian tarragon, silky wormwood, tarragon sagewort. Some of the foreign names are Estragon (Ger.), dragon, estragon (Fr.), dragoncillo, estragão (Sp.), estragão (Port.), dragon, dragon, long hao (Chin.), pelyněk kozalec (Czech.), połyń estragon (Russ.), tárkony üröm (Hung.), and vaistinis kietis (Georg.) (GBIF.org (2020); The Plant List, 2013; Catalogue of Life, 2020; Missouri Botanical Garden, 2020). The raw materials are dried *A. dracunculus* aerial parts and leaves–*Dracunculi herba* and *Dracunculi folium*, with an intense, aromatic fragrance (Food and Drug Administration, 2020).

*A. dracunculus* is a hairless perennial, reaching a height of up to 150 cm. Its straight stems are ribbed and have no flowers in the lower parts. The leaves are arranged alternately, sessile. The lower leaves are tripartite at the apex, while the middle and upper leaves are lanceolate. The tip of the leaf is sharp and the leaf blade margins entire. Yellow, tubular flowers are gathered in hanging, spherical capitula forming loose panicles. The fruit are achenes. The plant has strong, woody rhizomes, 0.5–1.5 cm thick, from which clusters of small roots grow (Aglarova et al., 2008; Bakova et al., 2017; Koul and Taak, 2017). *A. dracunculus* originates from areas of Siberia and Mongolia (Aglarova et al., 2008). In its natural habitats, this species can be found in Central Asia, in Mediterranean countries, in Eastern Europe and in North America. *A. dracunculus* grows in meadows in alkaline soils, in birch forests, near rivers, on mountain slopes and steppes (Aglarova et al., 2008).

*A. dracunculus* is a plant widely cultivated in the Americas, Asia and Europe. Two varieties are usually grown on plantations: French tarragon, otherwise known as true tarragon, and German tarragon (*Artemisia dracunculus* var. *sativa*). Russian tarragon is also found among the cultivated plants (miscellaneous varieties, including *A. dracunculus* var*. dracunculoides,* and *A. dracunculus* var*. inodora*). It is not popular, however, because of the poorer taste and less intense fragrance of the leaves (Eisenman et al., 2011; Obolskiy et al., 2011; Watson and Kennel, 2014).

*A. dracunculus* has low requirements in terms of cultivation site and care, but the highest yields are obtained from crops growing on moist, sandy-clay soils with an alkaline reaction. This species, depending on the cultivar, can be propagated vegetatively or from seed (Russian tarragon), or solely vegetatively from rhizome cuttings (French and German tarragon). It is also possible to use the *in vitro* micropropagation protocols developed for this species–described later in this review. In European conditions, plantations are established in april. The cuttings are placed in rows spaced at 60 cm and covered with a thin layer of soil. The first harvest of the herb takes place in dry weather in the same year, while in the following years two-to-three harvests can be gathered per year. The collected raw material is dried in drying sheds with natural air circulation or heated to 35°C. After drying, the leafy parts of the tarragon plant are separated from the hard stems (Aglarova et al., 2008; Eisenman et al., 2011; Obolskiy et al., 2011; Watson and Kennel, 2014).

**FIGURE 1:**
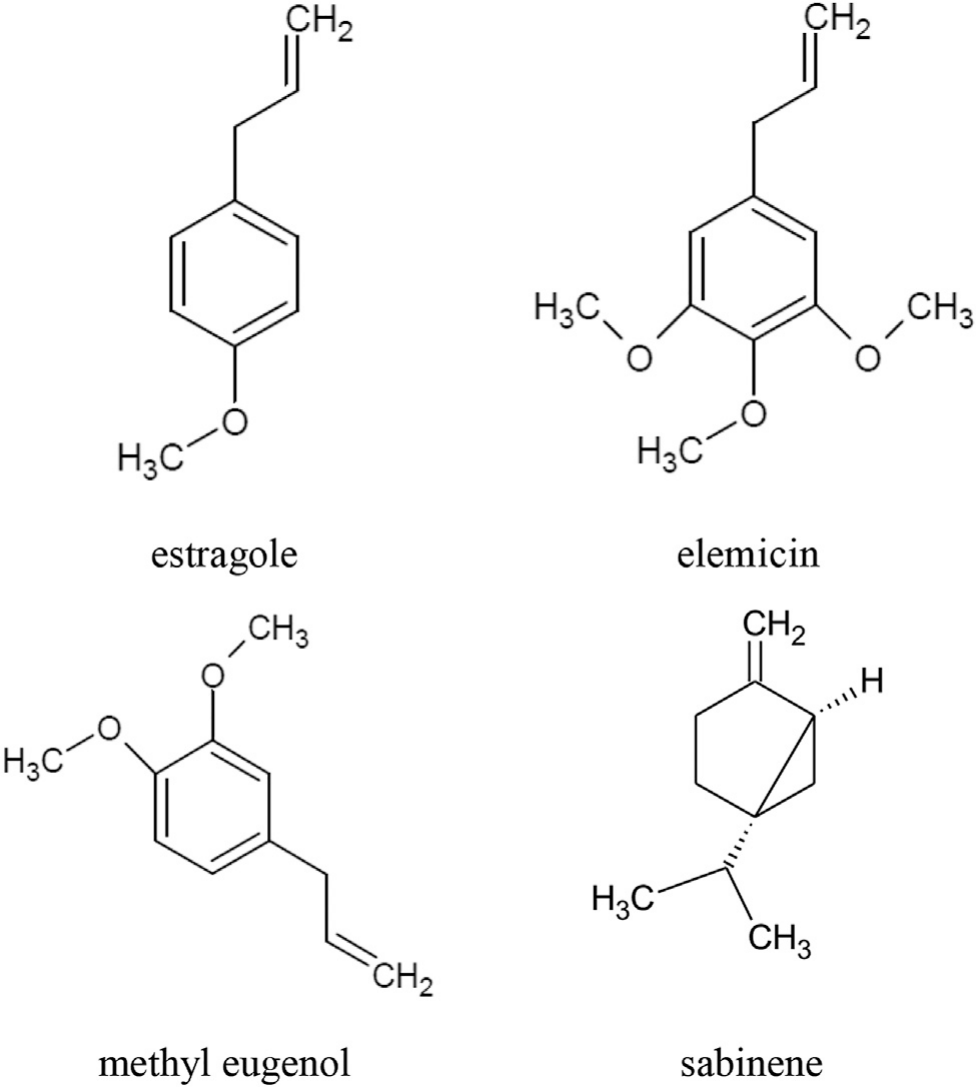
Chemical structure of volatile compounds characteristic of the essential oil of the *A. dracunculus* herb.

The main component of the raw materials, i.e. herb and leaves, is essential oil. The composition of *A. dracunculus* essential oil depends, *inter alia*, on the location of the cultivation site, the salinity of the soil and the age of the plant. The highest concentrations of the essential oil are observed at the beginning of leaf budding and at the beginning of flowering. The main components of the essential oil are: estragole, otherwise known as methyl chavicol or *p*-allylanisole (40–85%), sabinene (approx. 35%), methyl eugenol (approx. 25%), and elemicin (up to 57%) (Figure 1). Other compounds present in the oil in concentrations greater than 10% are: terpinen-4-ol, *β*-ocimene, *cis*-ocimene, *α*-trans-ocimene, limonene and *trans*-anethole, *α-*phellandrene, *β*-phellandrene (*Z*)-artemidin, capillene (Table 1) (Aglarova et al., 2008; Ayoughi et al., 2011; Obolskiy et al., 2011; Tak et al., 2014; Karimi et al., 2015; Abdollahnejad et al., 2016; Hassanzadeh et al., 2016; Joshi et al., 2016; Bedini et al., 2017; Sharopov et al., 2020). Phenylpropanoids (73.5%) constitute the main group of essential oil compounds. Monoterpenoids (24.3%) and sesquiterpenoids (0.2%) are present in the oil in considerably smaller amounts (Talbi et al., 2016; Bedini et al., 2017).

**TABLE 1:** Chemical composition of *A. dracunculus* essential oil.

Compounds	References
Phenylpropane derivatives	
Estragole (methylchavicol, *p*-allylanisole)	Ayoughi et al. (2011), Obolskiy et al. (2011), Obistioiu et al. (2014), Tak et al. (2014), Karimi et al. (2015), Abdollahnejad et al. (2016), Abtahi Froushani et al. (2016), Behbahani et al. (2017), Osanloo et al. (2017), Bussmann et al. (2020), Sharopov et al. (2020)
Methyl eugenol	Ayoughi et al. (2011), Obolskiy et al. (2011), Obistioiu et al. (2014), Tak et al. (2014), Karimi et al. (2015), Abdollahnejad et al. (2016), Abtahi Froushani et al. (2016), Behbahani et al. (2017), Osanloo et al. (2017), Szczepanik et al. (2018), Bussmann et al. (2020), Sharopov et al. (2020)
Elemicin	Ayoughi et al. (2011), Obolskiy et al. (2011), Szczepanik et al. (2018), Bussmann et al. (2020)
Isoelemycin	Obolskiy et al. (2011), Szczepanik et al. (2018)
Eugenol	Ayoughi et al. (2011), Osanloo et al. (2017), Bussmann et al. (2020)
Isoeugenol methyl ether	Szczepaniket al. (2018), Socaciu et al. (2020)
Asarone, isoeugenol methyl *trans*-anethole	Obolskiy et al. (2011)
(*Z*)-Anethole	Ayoughi et al. (2011), Obolskiy et al. (2011), Bussmann et al. (2020)
Prestragol	Talbi et al. (2016)
Anethole, carpacin	Abdollahnejad et al. (2016)
Dillapiole	Ayoughi et al. (2011), Behbahani et al. (2017)
3-(*p*-Methoxyphenyl)-1,2-propanediol	Güvenalp et al. (2017)
Monoterpenoids	
Sabinene	Aglarova et al. (2008), Ayoughi et al. (2011), Obolskiy et al. (2011), Karimi et al. (2015), Bedini et al. (2017), Behbahani et al. (2017), Osanloo et al. (2017), Bussmann et al. (2020), Sharopov et al. (2020), Socaciu et al. (2020)
*cis allo*-Ocymen, *cis allo*-ocymen hydrate, *trans*-sabinene acetate, ethyl geranyl	Obolskiy et al. (2011), Bussmann et al. (2020)
*α*-Fenchene, *cis*-sabinene hydrate, camphor, geranyl acetate, (*E*)-*β*-ionone	Sharopov et al. (2020)
*γ*-Terpinene	Ayoughi et al. (2011), Obolskiy et al. (2011), Bedini et al. (2017), Osanloo et al. (2017), Szczepanik et al. (2018), Sharopov et al. (2020), Socaciu et al. (2020)
*α*-Terpinene	Ayoughi et al. (2011), Obolskiy et al. (2011), Bedini et al. (2017), Osanloo et al. (2017), Szczepanik et al. (2018), Sharopov et al. (2020)
*α*-Terpineol	Aglarova et al. (2008), Bedini et al. (2017), Szczepanik et al. (2018)
4-Terpineol	Obolskiy et al. (2011), Szczepanik et al. (2018), Sharopov et al. (2020)
Terpinolene	Aglarova et al. (2008), Ayoughi et al. (2011), Obolskiy et al. (2011), Karimi et al. (2015), Bedini et al. (2017), Szczepanik et al. (2018), Bussmann et al. (2020), Sharopov et al. (2020), Socaciu et al. (2020)
*α*-Terpinolene	Behbahani et al. (2017), Osanloo et al. (2017)
Linalool	Ayoughi et al. (2011), Obolskiy et al. (2011), Bedini et al. (2017), Behbahani et al. (2017), Osanloo et al. (2017), Szczepanik et al. (2018), Sharopov et al. (2020)
Limonene	Aglarova et al. (2008), Ayoughi et al. (2011), Obolskiy et al. (2011), Karimi et al. (2015), Bedini et al. (2017), Behbahani et al. (2017), Osanloo et al. (2017), Szczepanik et al. (2018), Bussmann et al. (2020), Sharopov et al. (2020)
*allo*-Ocimene	Ayoughi et al. (2011), Obolskiy et al. (2011), Bedini et al. (2017), Behbahani et al. (2017)
*trans-allo*-Ocimene	Bussmann et al. (2020), Sharopov et al. (2020)
*cis-β*-Ocimene	Joshi et al. (2016), Sharopov et al. (2020), Socaciu et al. (2020)
*trans β*-Ocimene	Aglarova et al. (2008), Sharopov et al. (2020), Socaciu et al. (2020)
Citronellol	Obolskiy et al. (2011), Szczepanik et al. (2018), Bussmann et al. (2020)
Citronellol acetate, neryl acetate	Szczepanik et al. (2018), Sharopov et al. (2020)
Citronellol formate, terpineol, *α-trans*-ocimene, *β-*ocimene	Obolskiy et al. (2011)
(*E*)-*β*-*o*-cymene *p*-mentha-1,3,8-triene	Szczepanik et al. (2018), Szczepanik et al. (2018)
*o*-Cymene	Osanloo et al. (2017), Socaciu et al. (2020)
*β*-Ocimene Y, allocimene, geranial, nerol, *β*-elemene, tricyclen	Osanloo et al. (2017)
4-Carene, *d*-limonene, 1,8-cineole, *trans*-4 thujanol	Socaciu et al. (2020)
Carvone	Osanloo et al. (2017), Szczepanik et al. (2018)
Myrcene	Ayoughi et al. (2011), Obolskiy et al. (2011), Bedini et al. (2017), Osanloo et al. (2017), Sharopov et al. (2020)
Phellandrene	Osanloo et al. (2017)
*α*-Phellandrene	Obolskiy et al. (2011), Behbahani et al. (2017), Szczepanik et al. (2018), Vervandier-Fasseur and Latruffe (2019), Sharopov et al. (2020), Socaciu et al. (2020)
*β*-Phellandrene	Ayoughi et al. (2011), Obolskiy et al. (2011), Joshi et al. (2016), Sharopov et al. (2020)
*α*-Thujene	Osanloo et al. (2017), Szczepanik et al. (2018), Bussmann et al. (2020), Sharopov et al. (2020)
*α*-Pinene	Aglarova et al. (2008), Ayoughi et al. (2011), Obolskiy et al. (2011), Karimi et al. (2015), Abdollahnejad et al. (2016), Bedini et al. (2017), Behbahani et al. (2017), Osanloo et al. (2017), Szczepanik et al. (2018), Bussmann et al. (2020), Sharopov et al. (2020)
*β*-Pinene	Aglarova et al. (2008), Ayoughi et al. (2011), Karimi et al. (2015), Behbahani et al. (2017), Osanloo et al. (2017), Szczepanik et al. (2018), Sharopov et al. (2020), Socaciu et al. (2020)
Camphene	Aglarova et al. (2008), Ayoughi et al. (2011), Obolskiy et al. (2011), Karimi et al. (2015), Bedini et al. (2017), Osanloo et al. (2017), Bussmann et al. (2020), Sharopov et al. (2020)
*p*-Cymene	Ayoughi et al. (2011), Szczepanik et al. (2018), Jahani et al. (2019), Bussmann et al. (2020), Sharopov et al. (2020)
*E-β*-Ocymene, *Z-β*-ocymene	Ayoughi et al. (2011), Obolskiy et al. (2011), Bedini et al. (2017), Behbahani et al. (2017)
*neo-allo*-Ocymene	Aglarova et al. (2008), Ayoughi et al. (2011)
Thymol	Aglarova et al. (2008), Ayoughi et al. (2011), Szczepanik et al. (2018)
*β*-Myrcene	Aglarova et al. (2008), Ayoughi et al (2011), Behbahani et al. (2017), Osanloo et al. (2017)
1,8-Cineol, isoterpinolene, artemisinic ketone, isobornyl acetate, pseudolimonene	Bedini et al. (2017)
2-*allo*-cimene, 2-*β*-pinene, endo-isofenchene, *trans*-carveol, *α*-fenchene	Abdollahnejad et al. (2016)
∆3-carene	Ayoughi et al. (2011), Abdollahnejad et al. (2016), Bedini et al. (2017), Szczepanik et al. (2018)
Borneol, *E*-carvone oxide	Ayoughi et al. (2011)
*β*-Sesquifelandrene	Karimi et al. (2015)
Bornyl acetate	Ayoughi et al. (2011), Karimi et al. (2015), Behbahani et al. (2017), Osanloo et al. (2017), Sharopov et al. (2020)
Geraniol, *p*-pinene, *trans*-ocimene	Bussmann et al. (2020)
Myrtenal, pinocarveol	Aglarova et al. (2008)
Carvacrol, *α*-terpenyl acetate	Aglarova et al. (2008), Szczepanik et al. (2018)
Sesquiterpenoids	
Spatulenol	Aglarova et al. (2008), Ayoughi et al. (2011), Obolskiy et al. (2011), Karimi et al. (2015), Abdollahnejad et al. (2016), Bedini et al. (2017), Behbahani et al. (2017), Osanloo et al. (2017), Bussmann et al. (2020)
Spathunelol	Osanloo et al. (2017), Szczepanik et al. (2018), Sharopov et al. (2020)
*α*-Humulene	Ayoughi et al. (2011), Karimi et al. (2015), Osanloo et al. (2017), Sharopov et al. (2020)
Germacrene-D-4-ol, *α*-himachalene	Obolskiy et al. (2011)
Germacrene D	Obolskiy et al. (2011), Karimi et al. (2015)
Farnesane	Bussmann et al. (2020)
ar-Curcumen, caryophyllene oxide, *α*-bisabolol, *β*-bisabolen	Aglarova et al. (2008)
*E*-Caryophyllene	Karimi et al. (2015), Osanloo et al. (2017)
*β-*Caryophyllene	Bedini et al. (2017), Sharopov et al. (2020)
Caryophyllene	Szczepanik et al. (2018)
*α*-Cedrene	Bedini et al. (2017)
Elemene	Abdollahnejad et al. (2016)
*E-β*-Caryophyllene, (*E,E*)-farnesane, gleenol, *α-epi*-cadinol	Ayoughi et al. (2011)
bicyclermacren, *δ*-elemene	Ayoughi et al. (2011), Karimi et al. (2015)
*α*-Zingiberene	Karimi et al. (2015)
*E,E-α*-Farnesane	Karimi et al. (2015), Osanloo et al. (2017), Sharopov et al. (2020)
*α*-Bergamotene, acoradiene, germacrene D, *cis*-trans-*α*-farnesene	Osanloo et al. (2017)
*α*-Copaene, (*E*)-*β*-farnesene	Osanloo et al. (2017), Sharopov et al. (2020)
*β*-Sesquiphellandrene	Osanloo et al. (2017), Sharopov et al. (2020)
*Γ-*Elemene, *ar*-curcumene, bicyclogermacrene, *δ*-cadinene	Sharopov et al. (2020)
Diterpenoids	
Phytol	Ayoughi et al. (2011), Karimi et al. (2015)
Triterpenoids	
Squalene	Obolskiy et al. (2011)
Polyacetylenes	
Capillene	Aglarova et al. (2008), Chauhan et al. (2010), Verma et al. (2010), Obolskiy et al. (2011), Joshi et al. (2016)
1-Phenyl-2,4-hexadiene, 1-phenyl-2,4-hexadiene-1-one	Aglarova et al. (2008)
Isocoumarins	
3-(1*-Z*-Butenyl) isocoumarin=(*Z*)-artemidin, 2-(1-*E*-butenyl)-isocoumarin =(*E*)-artemidin	Aglarova et al. (2008), Sharopov et al. (2020)
Other compounds	
Dehydro-1,8-cineole, myrysticin	Szczepanik et al. (2018), Sharopov et al. (2020)
Apiole	Szczepanik et al. (2018)
Acenaphthene	Joshi et al. (2016), Osanloo et al. (2017)
3-Methoxycinnamaldehyde, *p*-allyphenol, cyclohexylmorpholine, cinnamic acid methyl ester, cinnamyl acetate, nonadecane	Osanloo et al. (2017)
5-Phenyl-1,3-pentadiyne	Joshi et al. (2016)
Hexanal	Socaciu et al. (2020)
1,3-Oktadiene, methyl salicylate, 1-pentadecene	Sharopov et al. (2020)

**FIGURE 2:**
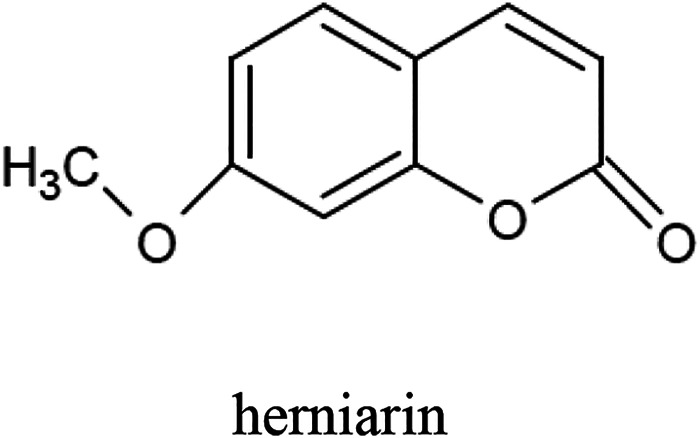
Chemical structure of herniarin–compound characteristic for the *A. dracunculus* herb.

The tarragon plant contains numerous coumarins, mainly herniarin (Figure 2), as well as coumarin, scopoletin, scoparone, dracumerin, artemidine, esculetin, esculin, and capillarin. The total amount of coumarins in the herb is over 1.0%. The biosynthesis of these compounds begins at an early stage of plant development; therefore, in three-month-old specimens, the coumarin content may even reach 1.3%. The maximum level of this group of compounds has been found in five-year-old plants (Aglarova et al., 2008; Bhutia and Valant-vetschera, 2008; Obolskiy et al., 2011; Talbi et al., 2016; Mumivand et al., 2017; Bussmann et al., 2020; Sharopov et al., 2020).

*A. dracunculus* has also been found to contain flavonoids, the concentration of which in wild plants varies between 0.5 and 1.9%. Under cultivation conditions, a maximum content of 4.9% can be obtained. Flavonoids typical of this species include quercetin, kaempferol, luteolin, isorhamnetin and their glycosides, naringenin, annagenin (5,6,7,8,4′-pentahydroxy-3′-methoxyflavone), pinocembrin and estragonoside C. *A. dracunculus* herb extracts have also been proven to contain phenolic acids, mainly chlorogenic acid, caffeic acid and vanillic acid. Other compounds found in the plant include alkamides (neopellitorine A, neopellitorine B, pellitorine), polyacetylenes, tannins, bitterness-imparting compounds, vitamin C, fatty acids and sterols, iodine compounds, and peroxidase (Aglarova et al., 2008; Mumivand et al., 2017; Jahani et al., 2019; Bussmann et al., 2020).

The chemical composition of *A. dracunculus* and the compounds contained in the plant’s essential oil are presented in Table 2.

**TABLE 2:** Chemical composition of *A. dracunculus.*

Group of compounds	Compounds	References
Flavonoids	2′,4′-Dihydroxy-4-methoxydihydrochalcone syn. DMC-2; 4-O-methyldavidigenin	Obolskiy et al. (2011), Yu et al. (2019), Majdan et al. (2020)
	Quercetin	Obolskiy et al. (2011), Mumivand et al. (2017), Jahani et al. (2019), Bussmann et al. (2020), Majdan et al. (2020)
	Kaempferol	Obolskiy et al. (2011), Bussmann et al. (2020)
	Luteolin	Obolskiy et al. (2011), Mumivand et al. (2017), Jahani et al., 2019, Bussmann et al. (2020)
	Apigenin	Mumivand et al. (2017), Majdan et al. (2020)
	Pinocembrin	Obolskiy et al. (2011), Mumivand et al. (2017), Jahani et al., 2019, Bussmann et al. (2020), Majdan et al. (2020)
	Naringenin	Obolskiy et al. (2011), Mumivand et al. (2017), Majdan et al. (2020)
	3,5,4-Trihydroxy-7,3′-dimethoxyflavone,	Obolskiy et al. (2011)
	3,5,4′-trihydroxy-7-methoxyflavone,	
	5,6,7,8,4′-pentahydroxymetoflavone,	
	5,7-dihydroxy flavone,	
	7-*O*-*β-* *d*-glycopyranoside,	
	5,7-dihydroxflavone,	
	7-*O*-*β-* *d*-glucopyranoside anangenin estragonizide	
	Davidigenin	Obolskiy et al. (2011), Yu et al. (2019), Majdan et al. (2020)
	Sacuranetine	
	Rutoside	Güvenalp et al. (2017)
	Quercetin 3-*O*-rutinoside	Ribeiro et al. (2016)
	Isoquercitrin	Majdan et al. (2020)
	Patuletin 3-*O*-malonylrobinobioside	
	Patuletin hexoside	
	Patuletin rhamnosylhexoside	
	Patuletin malonylrhamnosylhexoside	
	Vicenin	
	7,3′-Dimethyleriodictyol	Bhutia and Valant-vetschera (2008)
	7-Methyleriodictyol	
	7-Methylaringenine	
	Biocovertsetin	Bussmann et al. (2020)
	Hyperoside	
	Rutoside	
	Estroside	Aglarova et al. (2008)
	Kaempferol glycosides	
	Quercetin glycosides	
	Luteolin glycosides	
	Isorhamnetin glycosides	Aglarova et al. (2008), Majdan et al. (2020)
Coumarins	Herniarin	Aglarova et al. (2008), Güvenalp et al. (2017), Mumivand et al. (2017), Osanloo et al., 2017, Jahani et al., 2019, Aydin et al., 2020, Bussmann et al. (2020)
	3,4-Dehydroherniarin, skimmin,	Aydin et al. (2020)
	(-)-(*R*)-20-methoxydihydro-artemidine,	Obolskiy et al. (2011)
	(+)-(*R*)-(*E*)-3′-hydroxyartemidine,	
	(+)-(*S,R*)-epoxyartemidine,	
	4-hydroxycoumarin,	
	7,8-methylenedioxy-6-methoxycoumarin,	
	8-hydroksyartemidin artemidiol artemidynal ether,	
	7-methyl daphnetin ether methylenedaphnetin,	
	isovalerate capillarin,	
	γ,γ-dimethylallyl ether esculetin	
	6-Demethoxycapilarisine	Obolskiy et al. (2011), Majdan et al. (2020)
	Dacumerin	Bhutia and Valant-vetschera (2008), Obolskiy et al. (2011)
	Scoparon	
	Scopoletin	Obolskiy et al. (2011), Bussmann et al. (2020)
	Arethinol	Bussmann et al. (2020)
	Aridiodiol	
	Artidin	
	Isocoumarin	
	9-Hydroxyartemidine, esculetin	Aglarova et al. (2008)
	8-Hydroxycapillarin, artemidinol	Aglarova et al. (2008), Obolskiy et al. (2011)
	Esculin	
	Capillarin	
	Artemidine	Aglarova et al. (2008), Bhutia and Valant-vetschera, 2008, Obolskiy et al. (2011)
	Coumarin	Aglarova et al. (2008), Jahani et al. (2019)
Phenolic acids	(*E*) 2-Hydroxy-4-methoxycinnamic acid,	Obolskiy et al. (2011)
	4,5-di-*O*-caffeoylquinic acid,	
	5-*O*-caffeoylquinic acid hydroxybenzoic acid,	
	3,5-*O*-dicaffeoylquinic acid, *p*-coumaroyl-feruloylquinic acid, *p*-coumaroyl-caffeoylquinic acid,	
	4,5-di-*O*-caffeoylquinic acid,	Obolskiy et al. (2011), Ribeiro et al. (2016)
	5-*O*-caffeoylquinic acid,	
	Ferulic acid hexoside	Ribeiro et al. (2016), Majdan et al. (2020)
	Caffeoylquinic acid, sakuranetin	Majdan et al. (2020)
	Chicory acid	Obolskiy et al. (2011), Mumivand et al. (2017)
	Caffeic acid, chlorogenic acid	Obolskiy et al. (2011), Mumivand et al. (2017), Jahani et al. (2019), Bussmann et al. (2020), Majdan et al. (2020)
	*p*-Coumaric acid	Mumivand et al. (2017)
	Ferulic acid, syringic acid	Mumivand et al. (2017), Jahani et al. (2019)
	Vanillic acid	Mumivand et al. (2017), Jahani et al., 2019, Bussmann et al. (2020)
	2-Methoxycinnamic acid	Abdollahnejad et al. (2016)
Alkamides	Neopelitorin A, neopelitorin B, pelitoryin	Obolskiy et al. (2011), Majdan et al. (2020)
Sterols	Stigmasterol	Aydin et al. (2020)
Fatty acids	Myristic acid, oleic acid, palmitic acid	Obolskiy et al. (2011)
Vitamins	Vitamin C	Aglarova et al. (2008), Obolskiy et al. (2011)
Minerals	Iodine compounds	Mohammadi et al. (2020)
Enzymes	Peroxidases	Aglarova et al. (2008)
Tannins	No data	Bussmann et al. (2020)
Other compounds	1-Methoxy-4-(2-propenyl) benzene, 3,7-dimethyl-1,3,7-octatriene	Obolskiy et al. (2011)
	4-(1′,1′,2′,2′-Tetramethylpropyl)-1,2-benzenediol	Güvenalp et al. (2017)
	Phytoalexin	Talbi et al. (2016)
	Benzyl benzoate, methyl salicylate, trimethoxyallylbenzene	Aglarova et al. (2008)
	1,9,2-Octalone, 7-methoxy-1-indanone, cinnamic aldehyde, simetyloacetal, ociminon acetate, 3- methylbenzyl	Abdollahnejad et al. (2016)
	Anisaldehyde	Abdollahnejad et al. (2016), Osanloo et al. (2017)
	*γ-*Decalactone	Ayoughi et al. (2011), Karimi et al. (2015)
	Cuminic aldehyde, 2(3H)-furanone,5-hexyldihydro-benzen, ethanol, α-2-propenyl-methyl cinnamate	Osanloo et al. (2017)
		Karimi et al. (2015), Behbahani et al. (2017)
	(2*E*,4*E*)-1-(Piperidin-1-yl)undeca-2,4-diene-8,10-diyn-1-one, (2*E*,4*E*)-*N*-isobutyl undeca-2,4-dien-8,10-diynamide	Aydın et al. (2019), Aydin et al. (2020)

## Importance in the History of Medicine and Pharmacy

According to Pliny the Elder (1st c. AD), the name *A. dracunculus* L., a diminutive of the Latin word “draco” – dragon (Gr. δράκων), dracunculus–a small dragon, was given to this plant because of its serpentine rhizomes (Plinii Secundii, 1845). It was supposed to protect against snakebite when carried on one’s body or imbibed as a drink, and its juice was used in ear diseases. The name *A. dracunculus* might also be a distorted version of the Arabic name for tarragon, i.e. tharchum, from which the synonyms tarchon, tarcon and torchun are derived (Rejewski, 1996).

The term “dracunculus” was often used by ancient authors, e.g. by Dioscorides (1st c. AD), to refer to another species–*Arum dracunculus* L. (Gr. Drakontaia megale, Δρακονταία μεγάλή), or *Arum maculatum* L. (Gr. Drakontaia mikre, Δρακονταία μικρή) (Pedanius, 1998). *A. dracunculus* L. was commonly called draco, e.g. the botanist and German physician Valerius Cordus (16th c.) uses the name Draco sativus. Due to the similarity of dracunculus leaves to those of flax, it was believed to grow from flax seeds embedded in a hollowed-out onion (*ex semine lini in cepe*), meaning that it did not grow naturally. Authors such as K. Gessner (Bibliotheca Universalis 1545), P. Matthiolus (Commentarii in sex libros Pedacii Dioscoridis Anazarbi de Medicina material 1570) and J. Dalechamps (Historia Generalis Plantarum 1586) did not agree with this view (Bauhin and Cherler, 1651).

*P. Matthiolus* (1501–1577) describes tarragon (German version Dragoncell, Dracuncellus, Dragoncellus, Dracunculus esculentus) with the following: sharp taste, warming effect, stimulating the appetite (int.), externally applied with saliva, leaves crushed, mixed with honey, smeared causes bruises to disappear (Avicenna calls it “tarcon”) (Mattioli, 1586).

In the 17th-century “Herbarium” by Simon Syrennivs (1613), *A. dracunculus* L. bears the Polish name “torchun”, besides Dracunculus hortensis, Dragoncellus esculentus and also Draconkraut and Dragoncello. Its leaves are described as “elongated as flax leaves”, and the taste as “very peppery” or “spicy”. The plant has drying, warming and stimulating properties, relieves toothache, “removes mucus from the head”, stimulates digestion and has a diuretic effect. Commonly, tarragon is used in place of lettuce or in salad with other “green cabbages”, or just with salt. As a spice, it restores the appetite, eaten with salad or meat (Syrennivs, 1613).

In Krzysztof Kluk’s plant dictionary (“Dykcjonarz roślinny”, 18th c.), the colloquial name “draganek” is given, with the information that it grows in gardens, has lanceolate leaves, tasting “very spicy and pleasant”, which strengthen the stomach, “are suitable for salads and seasoning dishes; the vinegar containing these leaves can be very useful on the table” (Kluk, 1805).

In the 18th and 19th centuries, the stimulating tarragon herb was used in Europe as a spice plant rather than a medicinal plant (*Reizend, mehr in der Küche als in der Medizin angewendet*), which is confirmed by pharmacopoeias and dispensatories of that time: Dispensatorium pharmaceuticum Brunsvicense (Brunsvig 1777), P. J. Bergius, Materia Medica e Regno vegetabili (Stockholm 1782), Pharmacopoea Hispanica (Madrid 1798), Pharmacopoea Wirtemberica (Stuttgart 1798), Codex Medicamentarius sive Pharmacopoea Gallica (Paris 1818), V. L. Brera, Riccettario clinico (Padova 1825). The information given in them is as follows: *Artemisia dracunculus* L., Kaisersalat, Dragunbeifuß, Dragonkel, Estragon, Serpentine: southern European plant, grown in gardens; use is made of the herb–Herba Dracunculi esculenti sive hortensis–with thin, narrow, lanceolate, green leaves, which have a weak spicy aroma and a sharp, pungent, slightly spicy flavor (Jourdan, 1829).

The 19th-century Real-Encyclopädie der gesamten Pharmazie (1886), apart from providing a morphological description, states that *A. dracunculus* (Dragun, Bertram) is used as a spice, especially in the form of tarragon vinegar (Acetum Dracunculi) (Vulpius, 1887). The recipe for it can also be found in the Pharmaceutical Encyclopedia by L. Rządkowski: Acetum Dracunculi–Tarragon vinegar: Herbae Dracunculi recentis concisae 100, Aceti Vini 1000, Ac. Salicylici 1 – digest for eight days, squeeze in a wooden press, heat to a temperature not exceeding 100°C, filter after a few days. Pour into small bottles, tightly cork, and store lying down (Rządkowski, 1937).

## Applications in Traditional Medicine Around the World

In traditional medicine, *A. dracunculus* is used in ailments of the digestive system, and as an appetite and digestive stimulant, especially when red meat is consumed in large quantities (Uhl and Strauss, 2000). Moreover, the *A. dracunculus* herb was used to accelerate the metabolism (Senderski, 2007). It was also used as an anesthetic for toothache, wounds and cuts (Mamedov et al., 2004). In Europe the plant’s uses also included constipation, intestinal cramps, ulcers and cancer (Obolskiy et al., 2011). In Arabic cultures, the species was used in the treatment of insomnia, gingivitis, foot and mouth disease and as an agent for masking the taste of medicines, while in Central Asia, including Russia, it was used to treat irritation, allergic rashes, gastritis, dyspepsia, dermatitis, and to promote digestion and improve appetite (Mamedov et al., 2004; Sharopov et al., 2020). In Azerbaijan, *A. dracunculus* was used as an anti-epileptic drug (Alakbarov, 2001). Indian traditional medicine–Ayurveda–relates that the species is effective in the treatment of helminthiasis, intestinal smooth muscle spasms, fever of various origins, and a good tonic, an immunostimulant and to regulates the menstrual cycle (Miller and Miller, 1998; Obolskiy et al., 2011). Native people of Himachal Pradesh and Kashmir use a paste from the leaves of *A. dracunculus* in the treatment of wounds on the legs of yaks and donkeys. Moreover, they use extract of tarragon for toothache, fever, dysentery, intestinal worms and stomach ache (Joshi et al., 2016).

## Applications in Modern Phytotherapy and Position in Global Medicine

*A. dracunculus* is not a pharmacopeial species. The use of the species in medicine is based only on traditional medicine, but the plant has been a frequent subject of research in many centers around the world, especially in Iran. New findings on the biological activity of extracts from the herb, leaves, and essential oil of this species–proven by scientific research conducted over the last 10 years–are presented below. The partially known mechanisms of action of *A. dracunculus* are presented in Table 3.

**TABLE 3:** Pharmacological properties of *A. dracunculus*.

Activity	Mechanism of action	References
Anti-bacterial and anti-fungal	Inhibition of the growth of *Staphylococcus aureus, Staphylococcus epidermidis, Micrococcus luteus, Bacillus subtilis, Bacillus cereus, Listeria monocytogenes, Streptococcus pyogenes, Streptococcus typhimurium, Escherichia coli, Klebsiella pneumoniae, Shigella flexneri, Shigella marcescens, Pseudomonas aeruginosa* and *Salmonella* sp. under the influence of the essential oil of the *A. dracunculus* herb	Abdollahnejad et al. (2016)
	*Staphylococcus aureus, Proteus* spp. and *Corynebacterium diphtheriae* colony growth inhibition after application of the essential oil	Tajbakhsh and Soleimani (2018)
	essential oil of *A. dracunculus* leaves hampers the growth of *Escherichia coli, Listeria monocytogenes, Salmonella enteritidis* and *Staphylococcus aureus* strains	Socaciu et al. (2020)
	*Staphylococcus aureus, Staphylococcus aureus MRSA* (methicillin resistant)*, Bacillus cereus, Micrococcus flavus, Listeria monocytogenes, Pseudomonas eruginosa, A.R Pseudomonas eruginosa, Salmonella typhimurium, Escherichia coli, A.R Escherichia coli, Enterobacter cloacae* colonies growth inhibition and bactericidal effect as well as inhibition of the growth of *Aspergillus fumigatus, Aspergillus versicolor, Aspergillus ochraceus, Aspergillus niger, Trichoderma viride, Penicillum funiculosum, Penicillium ochrochloron, Penicillium verrucosum* and fungicidal activity under the influence of hydro-ethanolic extract of tarragon	Ribeiro et al. (2016)
	hydro-ethanolic extract of *A. dracunculus* leaves significantly reduces the number of colony-forming units (CFU) of *Candida albicans* in the liver and kidneys of mice	Zarasvand et al. (2016)
	Inhibition of the growth of bacteria: *Pseudomonas aeruginosa, Proteus vulgaris, Escherichia coli, Bacillus cereus, Bacillus subtilis, Staphylococcus aureus, Streptococcus pyogenes,* and fungi: *Aspergillus fumigatus, Penicillium expansum* and *Candida albicans*, under the influence of hydro-ethanolic herbal extract	Behbahani et al. (2017)
	Inhibition of the growth of bacteria: *Staphylococcus aureus*, *Staphylococcus epidermis, Staphylococcus aureus MRSA, Corynebacterium diphtheriae* and *Helicobacter pylori* after the application of infusion of *A. dracunculus* and minimal inhibition effect in *Enterococcus hirae, Klebsiella pneumoniae colonies*	Majdan et al. (2020)
Anti-protozoal	Inhibition of the development of the promastigote form of *Leishmania major*	Mirzaei et al. (2016)
Antioxidant	Reducing properties of the hydro-ethanolic herbal extract related to the presence of phenolic compounds and flavonoids	Behbahani et al. (2017), Ribeiro et al. (2016), Mumivand et al. (2017)
	Reduction of DPPH and ABTS in the presence of phenolic compounds	Zarezade et al. (2018)
Anti-inflammatory and analgesic	Reduction of pain sensations and reduction of xylene-induced ear edema after administration of the ethanolic herbal extract to mice	Eidi et al. (2016)
	Inhibition of ROS, IL-8 and TNF-α production in imitated inflammation	Majdan et al. (2020)
Immuno-modulating	Reduction in IL-17 and IFN-γ production and intensification of the phagocytosis process carried out by macrophages	Abtahi Froushani et al. (2016)
	Lowering of IL-17 and IL-23 levels and reduces the infiltration of leukocytes into brain cells	Safari et al. (2019)
	Increased neutrophil levels and decreased lymphocyte levels after intraperitoneal administration of the hydro-ethanolic extract from the leaves	Modaresi et al. (2018)
Anti-depressive	Increased resistance to stressful situations and reduction of stress-related levels of inflammatory cytokines	Wang et al. (2018)
	The phenolic compounds and flavonoids contained in the *A. dracunculus* herb reduce the immobility response time in mice in the writhing test and in the forced swim test	Jahani et al. (2019)
	Mild inhibition of hMAO-A and hMAO-B by extracts of *A. dracunculus*	Aydin et al. (2020)
Anti-tumor	Inhibition of proliferation of mouse lymphoma cells (L5178YD) due to the presence of polyphenols and alkamides in leaf extracts	Navarro-Salcedo et al. (2017)
Hepato-protective	Decrease in levels of alanine transaminase, aspartate transaminase, alkaline phosphatase and total bilirubin, and increase in total protein levels	Zarezade et al. (2018)
Hypo-glycaemic	Decrease in glycated hemoglobin, decrease in area under curve for insulin, decrease in total insulin secretion, decrease in systolic blood pressure, and increase in HDL-C	Méndez-Del Villar et al. (2016)
Normalizing the profile of thyroid hormones	Increase in thyroxine and triiodothyronine levels, decrease in elevated levels of thyrotropin, and increase in total antioxidant capacity, increase in glutathione, and decrease in malondialdehyde levels	Mohammadi et al. (2020)
Inhibiting the activity of carbonic anhydrase I and II	Compounds contained in herbal extracts reduce the activity of carbonic anhydrase I and II.	Aydın et al. (2019)
Repelling insects	Inhibition of *Calliphora vomitoria* egg laying on fresh beef, on which the essential oil of *A. dracunculus* herb was applied	Bedini et al. (2017)
	Larvacidal effect against *Anopheles stephensi* under the influence of nanoemulsion of *A. dracunculus* essential oil	Osanloo et al. (2017)

## Biological Activity Confirmed by Scientific Research

### Antibacterial and Antifungal Activities

Abdollahnejad et al. conducted a comparative study of the antibacterial potential of *A. dracunculus* herb oil obtained from two different methods: steam distillation and experimentally modified steam distillation. The experiment was carried out using the disk diffusion method and the microdilution method against *Staphylococcus aureus, S. epidermidis, Micrococcus luteus, Bacillus subtilis, B. cereus, Listeria monocytogenes, Streptococcus pyogenes, S. typhimurium, Escherichia coli, Klebsiella pneumoniae, Shigella flexneri, S. marcescens, Pseudomonas aeruginosa* and *Salmonella* spp. All these bacteria were found to be sensitive to the essential oil of *A. dracunculus*, with *S. epidermidis* showing the largest zone of inhibition (21.5 mm). The MIC value for Gram-positive bacteria did not differ significantly between oils obtained from the different methods, but a significantly lower MIC (minimum inhibitory concentration) value for Gram-negative bacteria was recorded for oil obtained with the modified steam distillation method (Abdollahnejad et al., 2016).

Two years later, a research team from the same facility conducted another experiment confirming the antibacterial activity of *A. dracunculus* oil against *Staphylococcus aureus, Klebsiella* spp.*, Salmonella typhimurium, Staphylococcus epidermidis, Proteus* spp. and *Corynebacterium diphtheriae*. The essential oil was tested with agar well diffusion. A significant inhibitory effect on the growth of *S. aureus, Proteus* spp. and *C. diphtheriae* bacterial strains was demonstrated. The MIC value for these bacteria was determined using the essential oil at a concentration of 0.03 and 25 mg/ml (Tajbakhsh and Soleimani, 2018).

Another study evaluating the antibacterial activity of *A. dracunculus* essential oil was conducted in 2020 by Socaciu et al. The experiment was aimed at assessment of the usability of the oil in antibacterial edible films. Bacteriostatic and bactericidal activities were evaluated with the Kirby-Bauer disk diffusion test, the minimum inhibitory concentration test (MIC) and the minimal bactericidal concentration (MBC) test. The results of the first test revealed the greatest inhibition of the growth of *Salmonella enteritidis* than *Staphylococcus aureus, Escherichia coli* and *Listeria monocytogenes.* The MIC and MBC tests displayed the highest bacteriostatic and bactericidal activity against *Escherichia coli* (5.14 MIC; 5.14 MBC) whereas in *Listeria monocytogenes* the bactericidal effect was poorer (5.14 MIC; 10.80 MBC) and lower values of MIC and MBC tests were obtained in *Salmonella enteritidis* (10.80 MIC; 10.80 MBC), and *Staphylococcus aureus* (10.80 MIC; 22.68 MBC) strains (Socaciu et al., 2020).

In 2016, conducted a study evaluating the antibacterial and antifungal activity of hydro-ethanolic extract of *A. dracunculus.* MIC and MBC tests along with the minimum fungicidal concentration test (MFC) were carried out to assess the antimicrobial activity of the extract. The experiment determined significant bactericidal activity of the extract and inhibition of the growth of *Staphylococcus aureus,* methicillin resistant (*MRSA*) *Bacillus cereus, Micrococcus flavus, Listeria monocytogenes, Pseudomonas aeruginosa,* antibiotic resistant (A.R) *Pseudomonas eruginosa, Salmonella typhimurium, Escherichia coli,* A.R. *Escherichia coli and Enterobacter cloacae* strains. *Bacillus cereus* being the most sensitive to the influence of hydro-ethanolic extract of tarragon (0.02 MIC; 0.08 MBC) followed by A.R *Pseudomonas aeruginosa* (0.04 MIC; 0.08 MBC) and *Enterobacter cloacae* (0.04 MIC; 0.08 MBC). Moreover, application of the extract in fungal colonies confirmed a notable decrement of the growth of the colonies and the fungicidal effect against *Aspergillus fumigatus, Aspergillus versicolor, Aspergillus ochraceus, Aspergillus niger, Trichoderma viride, Penicillum funiculosum, Penicillium ochrochloron, Penicillium verrucosum.* The MIC test results didn’t differ significantly in different colonies. Interestingly, *Aspergillus versicolor and Aspergillus niger* had lower responses in the MFC test (0.16) compared with the remaining fungi species (0.08) (Ribeiro et al., 2016).

The effect of hydro-ethanolic extract of *A. dracunculus* leaves on *C. albicans* infection was investigated. The experiment was carried out on an animal model (mouse). The rodents were treated intraperitoneally with the plant extract in doses of 50, 100, 200 mg/kg, then they were infected with 0.2 ml of a suspension at a concentration of 10^5^ colony-forming units per millilitre (CFU/ml). After sacrificing the animals, the concentration of the pathogen in liver and kidney homogenates was determined. It was found that the growth of *C. albicans* was significantly inhibited. For the maximum dose of the extract – 200 mg/kg, the concentration of the pathogen in the liver was 16.08 colony-forming units per gram of test material (CFU/g), and no traces of its presence were found in the kidneys. The amount in the control was 36.28 CFU/g and 53.31 CFU/g, respectively (Zarasvand et al., 2016).

In the other study, used the disk diffusion method, the pour-plate method and the dilution method to investigate the antibacterial and antifungal activities of a spice produced from *A. dracunculus* against strains of the bacteria: *Pseudomonas aeruginosa, Proteus vulgaris, Escherichia coli, Bacillus cereus, B. subtilis, Staphylococcus aureus, Streptococcus pyogenes,* and the fungi: *Aspergillus fumigatus, Penicillium expansum* and *Candida albicans*. At a concentration of 20 mg/ml, a hydro-ethanolic extract from the spice showed antimicrobial activity in the disk diffusion method. The largest zone of growth inhibition was observed for *S. pyogenes* (18 mm), and the smallest for *P. aeruginosa* (9 mm). In the pour-plate method, the extract at a concentration of 2 mg/ml was effective against *S. pyogenes, S. aureus, B. subtilis, B. cereus, C. albicans* and *A. fumigatus*. The extract showed antibacterial activity, but did not completely inhibit the growth of *E. coli* and *P. expansum*, and was ineffective against *P. vulgaris* and *P. aeruginosa*. In the microdilution method, the MIC value of the tarragon extract ranged from 2 to 32 mg/ml (Behbahani et al., 2017).

Majdan et al. investigated the antibacterial effects of aqueous extract of *A. dracunculus.* The study was conducted to evaluate the antibacterial activity of an infusion of aerial parts of tarragon against Gram-positive bacteria: *Staphylococcus aureus*, *S. epidermis, Corynebacterium diphtheriae, Enterococcus hirae* and Gram-negative bacteria: *Klebsiella pneumoniae, Escherichia coli, Proteus vulgaris* and *Helicobacter pylori* colonies*.* The values of concentrations of the extracts used in the assay ranged from 0.004 to 94.000 mg/ml (mg-relates to dry extract of *A. dracunculus* and mL relates to sterile distilled water). The results demonstrated that tarragon infusion was particularly effective against *Staphylococcus aureus* (MIC 0.09 mg/ml), to a lesser extent it also impeded growth of *Staphylococcus epidermis* (MIC 0.363 mg/ml)*, Corynebacterium diphtheriae* (MIC 5.9 mg/ml) colonies, *Staphylococcus aureus MRSA* (MIC 2.35 mg/ml) and *Helicobacter pylori* (MIC 11.75 mg/ml) colonies*.* On the contrary, minimal antimicrobial activity was displayed in *Klebsiella pneumoniae* (MIC 47 mg/mL) and *Enterococcus hirae* (MIC 23.5 mg/ml). Notably, *Escherichia coli* and *Proteus vulgaris* strains turned out to be invulnerable to the antimicrobial activity of the infusion (Majdan et al., 2020).

### Antiprotozoal Activity

Iranian researchers have investigated the potential of hydro-ethanolic extract of *A. dracunculus* in the treatment of leishmaniasis. They tested the effectiveness of various concentrations of the extract (100–1000 μg/ml) by applying them to the promastigote forms of *Leishmania major* grown *in vitro*. The recorded MIC values of the extract after 24, 48 and 72 h were: 962.03, 688.36 and 585.51 μg/ml, respectively, which indicates that the plant extract can be used in the treatment of leishmaniasis (Mirzaei et al., 2016).

### Antioxidant Effect

The antioxidant potential of *A. dracunculus* was assessed. For this purpose, fresh tarragon, purchased from the local market, was subjected to extraction with water and ethanol. The extract underwent a DPPH test and was used to determine total amounts of phenols and flavonoids by the spectrophotometric method. The estimated total phenolic content was 24.1 mg/g dry weight (as gallic acid eq.) and the total flavonoid content was 20 mg/g dry weight (as quercetin eq.). In the DPPH test, the IC_50_ value was 65.5 μg/ml, which confirms that *A. dracunculus* extracts can produce antioxidant effects (Behbahani et al., 2017).

The antioxidant activity has also been confirmed by other study where performed DPPH and ABTS tests and assessed the total phenolic content, thus verifying the activity of the hydro-ethanolic extract from the *A. dracunculus* herb. The total amount of phenols, expressed as gallic acid equivalents, was 197.22 mg/g dry weight. High activity of the extract was also demonstrated in the DPPH and ABTS tests (Zarezade et al., 2018).

The antioxidant activity of hydro-ethanolic extract of tarragon was also evaluated. First, Ribeiro et al. analyzed the phenolic content of the extract with HPLC. The results showed 147.5 mg of the phenolic content in 1g of the dried product including approximately 31.9 mg/g of flavonoids and 115.6 mg/g of phenolic acids. Then the team used different methods of assessing the antioxidant activity: the DPPH test examining radical scavenging activity, the β-carotene/linoleate test, reducing power was measured by the capacity to convert Fe^3+^ to Fe^2+^ and the TBARS assay evaluating the level of lipid peroxidation. Ribeiro et al. used ascorbic acid as a positive control. The results displayed that the extract is most effective as a reducing agent (Ribeiro et al., 2016).

In 2017, the researches from Iran and USA together also carried out DPPH tests, in addition to the ferric reducing antioxidant power (FRAP) test. For the experiment, hydro-methanolic extracts were used, which were prepared from the herb of *A. dracunculus* collected from various parts of Iran. The results showed that the antioxidant potential depended on the region from which the harvested plants originated. It was positively correlated with the concentration of compounds with phenolic and flavonoid structures. The highest reducing capacity was proven for extracts from the *A. dracunculus* herb collected in Birjand (a city in eastern Iran) – in the DPPH test the IC_50_ value was 0.039 mg/ml; in the FRAP test the extract was reduced 148.29 μmol Fe^2+^/g dry weight. The total amount of phenols calculated as gallic acid equivalents was 96.52 mg/g dry weight, and the total amount of flavonoids calculated as rutoside equivalents was 50.4 mg/g dry weight (Mumivand et al., 2017).

### Anti-inflammatory and Analgesic Effects

The Iranian research centers have investigated the antinociceptive and anti-inflammatory potential of an ethanolic extract from the *A. dracunculus* herb. The potential analgesic effect was verified in an animal model (mouse) using the hot plate test, the writhing test and the formalin test. Anti-inflammatory activity was assessed in a xylene-induced ear edema model. The study group received intraperitoneally the herbal ethanolic extract in doses of 5, 10, 50, or 100 mg/kg BW (body weight), while the control group was intraperitoneally administered a saline solution. Reduction in pain sensation was observed in all three tests. In the hot plate test, the extract administered in doses of 50 and 100 mg/kg increased the pain threshold after one hour. Interaction with opioid receptors may be responsible for the analgesic effect of the plant extract, as administration of naloxone to the animals reduced the antinociceptive effect of the extract. Anti-inflammatory activity has also been confirmed a significant reduction in ear edema was demonstrated with the extract administered in doses of 50 and 100 mg/kg (Eidi et al., 2016).

A study carried out by Majdan et al. mentioned above, evaluated the anti-inflammatory activity of aqueous extract of *A. dracunculus.* In the experiment researchers used neutrophils derived from venous peripheral blood from healthy human donors. Thereafter, to assess the secretion of cytokines after the incitement of neutrophils, an enzyme-linked immunosorbent assay (ELISA) was used. The application of aqueous extract of tarragon produced a decrement of the release of IL-8 (by 4.0 and 4.8%) and TNFα (by 7.8 and 5.2%). Moreover, ROS production was also measured. It was evaluated by microplate reader which displayed an inhibition of ROS production by 1.4% (Majdan et al., 2020).

### Immunomodulatory Action

An experiment was conducted on laboratory animals (mice) to evaluate the immunomodulatory properties of an aqueous extract of *A. dracunculus* herb. The mice were immunized intraperitoneally with sheep erythrocytes then orally administered an aqueous extract of the *A. dracunculus* herb. An increase in the level of antibodies to sheep erythrocytes and a decrease in cellular immunity were documented. The treatment was also shown to reduce the production of pro-inflammatory agents–IL-17 and IFN-γ, and to increase the phagocytic potential of macrophages. The authors of the study concluded that an aqueous extract of the *A. dracunculus* herb could be a good immunomodulating agent; moreover, it was free from potentially harmful estragole and methyl eugenol (Abtahi Froushani et al., 2016).

The studies on the potential use of aqueous extract of *A. dracunculus* in the treatment of multiple sclerosis were performed, too. The experiments were carried out on mice in which autoimmune encephalomyelitis (EAE) was induced with the myelin oligodendrocyte glycoprotein. This model is an experimental animal model for multiple sclerosis. It has been proven that giving rodents aqueous extract of *A. dracunculus* significantly alleviates the symptoms of the disease. By using the iron (III) reduction method, an increase in the antioxidant potential was verified. The use of the extract also reduced the level of inflammatory cytokines (IL-17 and IL-23) and the infiltration of leukocytes into brain cells. The results of the study indicate that the compounds contained in *A. dracunculus* can potentially be used in the treatment of multiple sclerosis (Safari et al., 2019).

Studies of Modaresi et al. determined the effect of hydro-ethanolic extract from *A. dracunculus* leaves on the hematological parameters of mice. The parameters assessed were levels of leukocytes, erythrocytes, lymphocytes, monocytes and neutrophils. It was demonstrated that intraperitoneal administration of the extract at a dose of 200 mg/kg significantly increased the level of neutrophils in the blood of rodents and reduced lymphocyte levels. There were no significant effects on the number of leukocytes, red blood cells or monocytes (Modaresi et al., 2018).

### Antidepressant Effect

Ethanolic extract of *A. dracunculus* was tested for its potential to increase mental resilience. The study was conducted on mice administered orally with extract of *A. dracunculus* shoots. A model of depression with repetitive stress caused by fear of social failure was tested by leaving the rodents in a cage with an aggressive individual for 10 min and checking their tendency to avoid contact. The treatment was shown to increase resistance to depression and to reduce the level of inflammatory cytokines associated with the presence of stress (Wang et al., 2018).

An experiment conducted in 2019 has confirmed the antidepressant activity of the species. Harvested herb of *A. dracunculus* was subjected to extraction with ethanol, then the activity of the extract was assessed on animals (NMRI mice and Swiss mice), by performing the forced swim test, the writhing test, and the open-field test. The results of the study showed a reduction in immobility time in the forced swim test (for the extract dose of 400 mg/kg the immobility time was 153.6 s, and was shorter compared to the control group, in which the immobility time was 202.3 s), a reduction in immobility time in the writhing test (for the extract dose of 200 mg/kg the period of immobility was 117.2 s, and was shorter compared to the control group, in which the immobility time was 142.6 s). In the open-field test, the rodents’ mobility did not change significantly, except for the trial with Swiss mice, which were administered 100 mg/kg of *A. dracunculus* herb extract.

The authors of the study associate the plant’s antidepressant activity with the presence of phenolic and flavonoid compounds, such as chlorogenic acid, caffeic acid or luteolin and quercetin (Jahani et al., 2019).

In 2020 scientists from Turkey carried out an experiment evaluating the influence of *A. dracunculus* extracts on human monoamine oxidase A (hMAO-A) and monoamine oxidase-B (hMAO-B). Isoenzymes are an important factor in the development of neurodegenerative diseases such as Alzheimer’s disease and Parkinson’s disease as well as in depression. Inhibitors of the enzymes have displayed efficiency in the treatment of neurodegenerative diseases and they are being used in treatment of clinical depression and anxiety. The teams prepared extracts of tarragon with different solvents: ethyl acetate, acetone, methanol and water to compare the activity of various types of extracts. Moreover, pure metabolites herniarin and skimmin were also tested to verify their influence on the isoenzymes. The results determined a nonselective and lower inhibitory activity of tarragon extracts and pure metabolites on hMAO-A and hMAO-B in comparison with reference inhibitors (Selegiline and Clorgyline). The most effective of the extracts proved to be the methanol extract. Interestingly, pure metabolites had lower inhibitory activity on hMAO-A and hMAO-B compared with extracts. In this regard, the researchers suggested that there are synergistic interactions between compounds of the extract (Aydin et al., 2020).

### Anti-Tumor Effect

Researchers from Mexico have assessed the effect of *A. dracunculus* leaf extract on the proliferation of mouse lymphoma L5178Y cells. Extraction of the plant material was performed with hexane, ethyl acetate, acetone, ethanol, acetonitrile and supercritical carbon dioxide (scCO_2_). Anti-tumor activity was assessed using a tumor growth inhibition test that included measuring ascitic fluid volume and the number of tumor cells after administration of the plant extract to mice. In the control group the tumor cell count was 17.969 × 10^6^, whereas in the group of mice receiving the acetonitrile extract from *A. dracunculus* leaves the cell count was 0.1 × 10^6^. Oral administration of the extract obtained with supercritical carbon dioxide reduced the number of cells to 12.9 × 10^6^, whereas intraperitoneal administration of the same extract reduced the number of cells to 0.1 × 10^6^. The anti-tumour activity of the acetonitrile extract is likely related to the high concentration of polyphenols, and the effect of the scCO_2_ extract is attributed to the presence of a higher concentration of alkamides in it (Navarro-Salcedo et al., 2017).

### Hepatoprotective Action

The hepatoprotective activity of a hydro-alcoholic extract of the herb of *A. dracunculus* was confirmed in 2018 as part of the cooperation between three research centers in Iran. In the course of the experiment, rats were given 50, 100, or 200 mg/kg of the extract for 15 days, followed by a single dose of carbon tetrachloride. Evidence was documented of a reduction in the levels of alanine transaminase, aspartate transaminase, alkaline phosphatase and total bilirubin, as well as a total protein increase. Histopathological examination also confirmed less liver damage in the group of animals given the herbal hydro-alcoholic extract (Zarezade et al., 2018).

### Hypoglycaemic Action

The effect of ethanolic extract of *A. dracunculus* herb on controlling glycaemia, insulin sensitivity and insulin secretion was tested. For this purpose, a randomized, double-blind clinical trial was conducted in 24 patients diagnosed with impaired glucose tolerance. Twice daily, the encapsulated ethanolic extract of *A. dracunculus* was administered at 1000 mg for 90 days. The documented results show a significant decrease in systolic blood pressure (120 mm Hg in the control group, 113 mmHg in the test group), a decrease in glycosylated hemoglobin concentration (5.8% in the control group, 5.6% in the test group), a decrease in the area under the curve for insulin levels (56.136–27.426 pmol/L in the control group, 44.472 to 23.370 pmol/L in the test group), and a reduction in the insulinogenic index (0.45–0.23 in the control group, 0.35 to 0.18 in the test group). HDL cholesterol levels increased. The results of the study showed that *A. dracunculus* herb extracts may in future be used as a therapeutic agent in the treatment of impaired glucose tolerance (Méndez-Del Villar et al., 2016).

### Thyroid Hormone Profile Regulation

The study assessed whether the *A. dracunculus* herb could be used in hypothyroidism. It was conducted on a group of forty-eight rats in which hypothyroidism was induced by the administration of propylthiouracil. The rodents were orally administered an aqueous extract of the herb. Samples of the animals’ blood were taken during the experiment. A significant increase in the level of thyroxine and triiodothyronine was proven after the administration of 300 mg/kg of the plant extract; moreover, a decrease in the elevated level of thyrotropin was recorded in the negative control group. At a dose of 200 mg/kg, the extract increased the total antioxidant capacity (TAC) and the level of glutathione. There was also a decrease in the level of malonaldehyde, a marker of oxidative stress. The research results indicate that *A. dracunculus* aqueous extract may improve the thyroid hormone profile, but further research is needed (Mohammadi et al., 2020).

### Inhibition of Carbonic Anhydrase I and II Activity

The study verified whether *A. dracunculus* herb extracts obtained with n-hexane, dichloromethane, ethanol and methanol are inhibitors of carbonic anhydrase I and II (hCA I and hCA II). In the body, these enzymes catalyze the reaction between water and carbon dioxide, which produces a proton and a bicarbonate anion. This reaction has a significant impact on the water content inside the eyeball. With excess fluid build-up, intraocular pressure rises, which can lead to the development of glaucoma. In the study, the highest activity (IC_50_ = 0.02 μg/ml for hCA I, and IC_50_ = 0.31 μg/ml for hCA II) was demonstrated for the dichloromethane extract. In order to determine the active compounds responsible for the action, the following main components were isolated from the dichloromethane extract: *trans*-anethole, stigmasterol, herniarin (2E, 4E)-N-isobutylundeca-2,4-diene-8,10-diynamide (2E, 4E)-1-(piperidin-1-yl)undeca-2,4-diene-8,10-diyn-1-one and 1-(4′-methoxyphenyl)-1,2,3-trihydroxypropane. All these compounds inhibited the activity of hCA I and hCA II. The action of 1-(4′-methoxyphenyl)-1,2,3-trihydroxypropane was more potent than that of the control acetazolamide. On the basis of their research, the authors of the study concluded that *A. dracunculus* herb extracts, like anhydrase I and II inhibitors, can reduce the accumulation of fluid inside the eyeball and thus be used in the treatment of glaucoma (Aydın et al., 2019).

### Insect-Repelling Action

Italian researchers have verified that the essential oil obtained from the herb *A. dracunculus* can act as a repellent against a species of dipterous flies–*Calliphora vomitoria* (the blue bottle fly). The insect, which is a synanthropic species, is responsible for the transmission of many pathogenic microorganisms–*Salmonella typhimurium, Entamoeba coli*, and *Giardia duodenalis*. Larvae that hatch from its eggs deposited on animal or human tissue cause myiasis. The essential oil of the plant has been shown to deter *C. vomitoria* from laying eggs in fresh beef. At an oil concentration of 0.05 μL/cm^2^, complete inhibition of egg laying by the insect was demonstrated (Bedini et al., 2017).

Osanloo et al. (2017) conducted a study confirming larvicidal activity of tarragon essential oil against *Anopheles stephensi*–mosquitos that are responsible for spreading malaria in the Arabian Peninsula, Indian subcontinent, Afghanistan and Iran. Chemical larvicides which are widely used to control the disease cause environmental pollution and desensitization of some species to active agents. Therefore, nanoemulsion of *A. dracunculus* essential oil was tested as a natural alternative to chemical products. The results showed that nanoemulsion consisting of 0.35% tarragon oil, 10% of Tween 20 and deionized water has a comparable larvicidal activity to chemical larvicides (Osanloo et al., 2017).

## Applications in Cosmetology

The European CosIng database allows the use of *A. dracunculus* in six forms (European Commission CosIng, 2020). The species is used in the cosmetics industry as an ingredient in skin care products, fragrances and masking agents. In cosmetology, *A. dracunculus* is used in the production of moisturizing creams, shampoos, lotions and cleansing milk. These preparations are used to care for the skin of the scalp, body and face (Table 4). The essential oil obtained by steam distillation is widely used as a component in perfumes (Aglarova et al., 2008). It is also used in aromatherapy during massages and baths and is added to facial masks and compresses (Hassanzadeh et al., 2016; Mumivand et al., 2017). Products containing tarragon are offered by both European and non-European cosmetics companies. Among them are brands such as the English *Lush Cosmetics*, the Italian *L'Erbolario Assenzio*, the German *AER Scent*, the French *Florame*, the Swedish *Timotei*, the Azerbaijani *Buta* and the South Korean *Missha*. As a component of women’s and men’s perfumes, the oil of *A. dracunculus* is very often used by prestigious fashion brands, such as the Italian *Prada, Versace, Dolce & Gabbana*, the French *Givenchy* and *Chloé*, the American *Calvin Klein* and *Tom Ford*, and many others.

**TABLE 4:** Applications of *A. dracunculus* in cosmetology as recommended by the CosIng database.

Name according to CosIng	Description	Application profile
*Artemisia dracunculus* flower	Flowers of the tarragon plant	Skin care agent
*Artemisia dracunculus* herb extract	Extract from tarragon herb	Fragrance
*Artemisia dracunculus* leaf/stem extract	Extract from tarragon leaves and stems	Masking agent
*Artemisia dracunculus* oil	Essential oil of tarragon	Fragrance, skin care agent
*Artemisia dracunculus* root extract	Extract from tarragon roots	Skin care agent
*Artemisia dracunculus* seed/*Anthemis nobilis* seed/*Hypericum androsaemum* seed extract	Extract from tarragon seeds, roman chamomile seeds, and tutsan (shrubby st. John’s wort) seeds	Skin care agent

## Applications in the Food Industry

The US Food and Drug Administration (FDA) states that the composition of the spice tarragon includes dried leaves and flowering tops of *A. dracunculus* (Food and Drug Administration, 1980). Tarragon is usually used as a seasoning for meat, sauces, rice dishes, fish and marinades. It has preservative properties, so it is keenly used for pickling cabbage and cucumbers, marinating pumpkins, and for the production of tarragon mustard and herbal vinegars. It is recommended for people on a salt-free diet because it improves the taste of dishes (Kordali et al., 2005; Méndez-Del Villar et al., 2016; Çorapcı et al., 2020). It is also added to infusions, refreshing drinks, alcoholic and non-alcoholic drinks such as “Tarkhun” – a carbonated, non-alcoholic drink, the main ingredient of which are fresh *A. dracunculus* leaves. Fresh tarragon leaves can be used as side dishes or garnishes with meat dishes and in vegetable salads (Goldstein, 1999; Ribnicky et al., 2004; Aglarova et al., 2008).

The nutritional composition of tarragon comprises a high content of carbohydrates (88g/100 g dried weight), lower amounts of fructose and sucrose as well as low levels of fat with a predominance of polyunsaturated fatty acids (Ribeiro et al., 2016). Use of the species in the food industry varies from country to country. Tarragon is a important herb in French cuisine. French tarragon or estragon comes from there and is the most popular variety of the spice for use in cooking. French tarragon has a refreshing, sweet and slightly bitter fragrance. The ground parts are used whole, both fresh and dried. After drying, it is milled or crushed. However, the fresh herb is more desirable, as dried herbs quickly lose their qualities. In France, tarragon is one of the key plants used in the production of Dijon mustard, cream sauces and mayonnaize. Armenians use tarragon to season vegetables, fish and meats. In Slovenia, tarragon is used to season the pastry called ‘potica’. In the United States, it is added to vinegar, tartar sauce and seafood (Goldstein, 1999; Ribnicky et al., 2004; Aglarova et al., 2008; Çorapcı et al., 2020).

The taste of *A. dracunculus* depends on the variety. French tarragon has a sweet taste and aroma similar to liquorice, with a slightly bitter aftertaste. Another description of French tarragon says that it has a herbaceous flavor similar to anise and basil. Russian tarragon is more bitter and pungent, devoid of the anise flavor (Uhl and Strauss, 2000).

Professional studies suggest that hydro-ethanolic extract of *A. dracunculus,* with its antimicrobial activity, can be considered a potential candidate for application in food as a preservative. Additionally, hydro-ethanolic extract is less toxic compared to essential oils, therefore, it has the potential to be used in the food industry. Furthermore, tarragon extract also displayed improvement in the fracture of pizza dough as well as a lower influence on dough darkening compared with ascorbic acid (Gottardi et al., 2016; Ribeiro et al., 2016).

A notable potential application of *A. dracunculus* essential oil is in edible antibacterial films used to prevention food spoilage. In 2020 a study by Socaciu and co-authors confirmed the antibacterial and antioxidant activity of tarragon oil. Moreover, the experiment also evaluated the influence of the oil on the qualities of edible antibacterial film. The results show that the application of essential oil in the film forming solution improved water solubility, protection against VIS light, puncture resistance, elasticity and transparency of the film and an increased of its moisture content. Therefore, tarragon oil can be considered an alternative to synthetic compounds in food packaging applications (Socaciu et al., 2020).

## Safety of Use

The FDA lists *A. dracunculus* and the oils and extracts derived from this species as safe for use (Food and Drug Administration, 2020). However, there have been reports of potential toxicity of the main components of the essential oil of the plant–estragole and methyl eugenol (Obolskiy et al., 2011). Methyl eugenol and estragole, as components of *A. dracunculus*, have both undergone extensive safety checks. Tests on animals administered estragole orally or subcutaneously have shown induction of liver tumors in mice. The appearance of tumors is associated with the formation of 1′-hydroxyestragole. This metabolite was also present in the urine of men who were given 100 μg of estragole for six months. Both compounds (estragole and 1′-hydroxyestragole) promoted abnormal DNA synthesis in rat hepatocytes, in both *in vivo* and *in vitro* tests (European Commission Health and Comsumer Protection Directorate - General, 2001).

Methyl eugenol has also been found to induce liver tumors in animal studies (rats and mice) and, additionally, the formation of neuroendocrine tumors in the glandular stomach. Neoplasms of the kidney, mammary glands and subcutaneous tissue, and mesotheliomas have also been documented in rats. High doses of methyl eugenol (minimum 30 mg/kg for 25 days) induce auto-induction of P450 cytochromes, leading to the formation of the carcinogenic 1′-hydroxymethyl eugenol. As in the case of estragole, methyl eugenol and its metabolites promote unplanned DNA synthesis; moreover, methyl eugenol forms DNA adducts (European Commission Health and Comsumer Protection Directorate-General, 2001).

After analyzing the available data, the European Food Safety Authority (EFSA) classified estragole and methyl eugenol as genotoxic and carcinogenic compounds. However, a safe threshold for their consumption has not been established. The Commission recommends limiting the use of both compounds (European Commission Health and Comsumer Protection Directorate - General, 2001).

The data of European Medicines Agency - The Committee on Herbal Medicinal Products (HMPC) from 2019 regarding the estragole use claimed that “there is the evidence of genotoxic carcinogenicity, exposure to this compound; estragole should be kept as low as practically achievable. In the evaluation of herbal medicinal products containing estragole Member States should take steps to ensure that the public are protected from exposure.” HMPC recommended the acceptable intake of estragole per day for adults - 51.8 mg/kg of body weight, and for children - 1.0 μg/kg of body weight. Moreover, report concluded, that the intake of estragole from (traditional) herbal medicinal products in the population should be as low as possible (European Medicines Agency - The Committee on Herbal Medicinal Products (HMPC), 2019).

## Plant Biotechnology Research

Due to the extensive use of tarragon in the food industry, large losses incurred with the traditional methods of its cultivation, difficulties in vegetative reproduction, and diseases of the cultivated plants, research is being conducted on alternative, biotechnological methods of propagating *A. dracunculus*.

In 2012, scientists developed an efficient protocol for micropropagation of the French variety of *A. dracunculus*. Plant cuttings 20 mm long were cut into 1 mm long pieces. These were then used to establish cultures in Murashige & Skoog (MS) liquid media, with five apical meristems as the experimental unit. In order to propagate the plant material, the established cultures were transferred to solid MS media. The tested propagation protocols differed from one another in the concentrations of indole acetic acid (IAA) and kinetin (KIN) added to the medium. The MS medium enriched with only 0.1 mg/L each of IAA and KIN, proved to be the most effective. The length of the sprouts was 11.19 mm and the multiplication index was 1.97. Thirteen days after planting, 100% rooting was achieved. As an alternative method of propagation, the authors of the study placed the propagating pieces horizontally on a solid MS medium supplemented with 0.5 mg/L KIN and 0.5 mg/L IAA. The sprout length was 10.31 mm and the multiplication index was 1.87; good callus induction and leaf development were also demonstrated. The time required to establish the *in vitro* culture was three weeks (Fernández-Lizarazo and Mosquera-Vásquez, 2012).

Türközü et al. also undertook to develop an *A. dracunculus* micro-propagation protocol. They proved that the highest efficiency (92%) could be obtained by growing cultures in MS medium supplemented with 1.8 µM 6-benzyladenine (BA) and 0.3 µM 1-naphthylacetic acid (NAA). They also reported an adverse effect of the addition of gibberellic acid (GA_3_) on microshoot development. The longest roots were obtained in the plants grown on MS medium with the addition of indolelbutyric acid (IBA) at a concentration of 0.5 mg/L (44 mm) and on ½ MS medium with 0.5 mg/L IBA (46 mm) (Türközü et al., 2014).

Another team of Ibrahim et al. has tested the performance of various explants–leaves, stems and roots of *A. dracunculus* in establishing *in vitro* cultures. Leaf explants placed on MS medium supplemented with 1.0 mg/ml of 2,4-dichlorophenoxyacetic acid (2,4-D) proved to be the best starting material for callus induction. For root and stem explants, no statistically significant effects were obtained. It was also proved that the best callus induction was caused by the addition of 0.5 mg/L BA and 0.1 mg/L NAA (75 shoots). It was also shown that the concentration of estragole in the cultured plants correlated with the type of explant and the phytohormone concentrations used. Estragole was not detected in the roots; the highest amount of it (16.7% estragole per g dry weight) was isolated from one-node cultures after the application of 0.5 mg/L 2,4-D (Ibrahim et al., 2011).

## Summary

*A. dracunculus* has been a frequent subject of research in the last few years, regarding both the chemistry and biological activity of extracts obtained from the herb and/or leaves, and the essential oil. Phytochemical tests have confirmed the presence of numerous flavonoid compounds, phenolic acids, coumarins and alkamides in the herb and leaves, as well as a very high variability of the chemical composition of the essential oil. Contemporary research on the biological activity of the above-mentioned raw materials has proven new findings in their activity–antibacterial, antifungal and antiprotozoal effects, as well as extremely valuable antioxidant, immunomodulatory and antineoplastic properties. These studies have also proven hepatoprotective, hypoglycaemic and thyroid-regulating effects. An antidepressant effect has also been documented. The anti-inflammatory and analgesic effects known from traditional medicine applications have also been confirmed.

The popularity of *A. dracunculus* in the production of cosmetics is also surprising. For this purpose, the essential oil of tarragon, extracts from the flowers, leaves, stems and seeds, as well as from the whole herb and roots are used. The essential oil is also used in aromatherapy treatments and in the production of perfumes. Cosmetics based on this species are offered by both European cosmetics companies (mainly English, German, French, Swedish) and non-European companies (mainly South Korean and Azerbaijani). The herb of the species is widely used for seasoning purposes and as an additive to alcoholic and non-alcoholic beverages. The species is the subject of research in the field of plant biotechnology, which mainly concerns the development of micropropagation protocols. The most valuable findings in the professional scientific research conducted has been the proof of new areas of the biological activity of the *A. dracunculus* herb and/or leaf extracts and essential oil–mainly their antioxidant, immunomodulating and antineoplastic effects, as well as the hepatoprotective and hypoglycaemic effects. The species, known thus far as a spice plant, appears to be an extremely valuable medicinal and cosmetic plant.
